# Emergence of a New Lineage 1C Variant of Porcine Reproductive and Respiratory Syndrome Virus 2 in the United States

**DOI:** 10.3389/fvets.2021.752938

**Published:** 2021-10-18

**Authors:** Mariana Kikuti, Igor A. D. Paploski, Nakarin Pamornchainavakul, Catalina Picasso-Risso, Mark Schwartz, Paul Yeske, Brad Leuwerke, Laura Bruner, Deborah Murray, Brian D. Roggow, Pete Thomas, Lori Feldmann, Matt Allerson, Melissa Hensch, Tyler Bauman, Brent Sexton, Albert Rovira, Kimberly VanderWaal, Cesar A. Corzo

**Affiliations:** ^1^Department of Veterinary Population Medicine, University of Minnesota, Saint Paul, MN, United States; ^2^Schwartz Farms Inc., Sleepy Eye, MN, United States; ^3^Swine Vet Center, St. Peter, MN, United States; ^4^New Fashion Pork, Jackson, MN, United States; ^5^Fairmont Veterinary Clinic, Fairmont, MN, United States; ^6^Iowa Select Farms, Iowa Falls, IA, United States; ^7^Protein Sources LLC, Mapleton, MN, United States; ^8^Holden Farms Inc., Northfield, MN, United States; ^9^The Maschhoffs LLC, Carlyle, IL, United States

**Keywords:** disease outbreak, porcine reproductive and respiratory disease virus, epidemiology, swine diseases, epidemics

## Abstract

We report an ongoing regional outbreak of an emerging porcine reproductive and respiratory syndrome virus (PRRSV2) variant within Lineage 1C affecting 154 breeding and grow-finishing sites in the Midwestern U.S. Transmission seemed to have occurred in two waves, with the first peak of weekly cases occurring between October and December 2020 and the second starting in April 2021. Most of cases occurred within a 120 km radius. Both orf5 and whole genome sequencing results suggest that this represents the emergence of a new variant within Lineage 1C distinct from what has been previously circulating. A case-control study was conducted with 50 cases (sites affected with the newly emerged variant) and 58 controls (sites affected with other PRRSV variants) between October and December 2020. Sites that had a market vehicle that was not exclusive to the production system had 0.04 times the odds of being a case than a control. A spatial cluster (81.42 km radius) with 1.68 times higher the number of cases than controls was found. The average finishing mortality within the first 4 weeks after detection was higher amongst cases (4.50%) than controls (0.01%). The transmission of a highly similar virus between different farms carrying on trough spring rises concerns for the next high transmission season of PRRS.

## Introduction

Porcine reproductive and respiratory syndrome (PRRS), a non-reportable swine disease, is characterized by reproductive failure and high pre-weaning mortality in breeding herds whereas pneumonia leading to poor growth and high wean-to-market mortality is seen in grow-finishing herds. It is a major cause of economic losses to the U.S. swine industry (1) and worldwide. It is caused by the porcine reproductive and respiratory syndrome virus (PRRSV), a single-stranded RNA virus that can be classified into two distinct species, *Betaarterivirus suid 1* (PRRSV1) and *Betaarterivirus suid 2* (PRRSV2) (2). PRRSV2 are further categorized according to Restriction Fragment Length Polymorphism (RFLP) types (3) or phylogenetic lineages (4) and sub-lineages (5) based on the open reading frame 5 (orf5) of the virus genome.

Since its first identification in the U.S., periodical emergence of PRRSV2 variants causing nation-wide epidemics have been reported, primarily described by their RFLP cut pattern. The most recent PRRSV2 epidemics due to a newly emerged virus occurred in 2014–2015 associated with an RFLP pattern 1-7-4 and was reported to cause high production losses (6, 7). Moreover, with the popularization of orf5 sequencing as an additional surveillance tool for PRRS, molecular epidemiology investigations of farm and system-level outbreaks have become increasingly common. With that, lineage and sub-lineage classification has gained traction in the past couple of years. A periodical turnover of the most prevalent lineages/sub-lineages has also been observed multiple times in the span of 9 years (5). Although the frequent emergence of a new variant is expected, its determinant factors are still largely unknown. Because PRRSV is a RNA virus, a relatively high mutation rate and rapid evolution is expected (8). Combined with diversity originating from genomic recombination and genome reassortment (9), interpretation of PRRSV phylogeny is complex, which often hinders molecular investigations of outbreaks.

During the fall of 2020, the simultaneous occurrence of farm-level PRRS outbreaks caused by unusually similar viruses at the orf5 level was reported by swine producers in the Midwestern U.S. Three main factors caused these farm-level outbreaks to quickly attract attention from the industry: (1) PRRSV with high orf5 nucleotide identities (>99%) were being detected in multiple apparently unrelated sites from different production systems; (2) this variant was affecting mainly growing pig sites and seemed different from variants historically detected in those production systems; and (3) reports from the field suggested that affected sites were experiencing extremely high production losses. Here, we report early findings of this ongoing PRRS outbreak caused by a PRRSV2 in a swine-dense region of the U.S.

## Methods

These farm-level outbreaks were reported to the Morrison Swine Health Monitoring Project (MSHMP) by production systems currently enrolled in the project. MSHMP is a producer-driven nationwide program that monitors infectious diseases occurrence in ~50% of the U.S. breeding herd (10). The project currently monitors 38 pig production systems and collect both retrospective and prospective PRRSV orf5 sequences generated by the systems as a result of their routine outbreak investigations through their own laboratories, or at the University of Minnesota (UMN), Iowa State University (ISU), South Dakota State University (SDSU), and Kansas State University (KSU) veterinary diagnostic laboratories (VDL). Through this project, all PRRSV orf5 sequences generated through participant's routine monitoring efforts in breeding, gilt developing units, growing and finishing pig herds are captured. The orf5 PRRSV sequences associated with these newly reported farm-level PRRS outbreaks were compared to 30,000 historical orf5 sequences from MSHMP participating pig production systems from 1998 to May 2021.

Sequences from the reported farm-level outbreaks of interest formed a genetic cluster, in which a case was defined by nucleotide identity of ≥98% between samples. Spatiotemporal description of the cases and a directional method was performed using adjacent directed time to assess the spatiotemporal interaction of the cases and calculate the average direction in which cases spread over the course of the study (11). Cases were classified into lineage/sub-lineage and RFLP patterns unless they had incorrect initial and stop codons or presence of ambiguities that would hinder a reliable classification. In order to evaluate if this genetic cluster represented the emergence of a new variant within its sub-lineage (1C), a phylogenetic tree with cases detected up to December 2020 was constructed using NextStrain (12). Since it is an industry standard, we were able to compare case orf5 sequences to 8,922 Lineage 1C orf5 sequences (i.e., 2,181 from the U.S. publicly available from GenBank; 1,804 from the UMN VDL identified in 2007-January 2021, and 4,937 from MSHMP). Sensitivity, specificity, positive predictive value and negative predictive value for RFLP pattern (1-4-4), lineage classification (Lineage 1C), and a combination of both in identifying these cases were assessed using the MSHMP sequence database for PRRSV2 sequences identified between January 2020 and May 2021. These two classifications specifically were chosen as they have been widely used in the field to describe these outbreaks. Briefly, true positive, false positive, true negative, and false negative rates were calculated for each classification based on their ability to identify the cases genetic cluster as defined above. Additionally, whole genome sequence (WGS) was obtained for a subset of 16 cases with available diagnostic specimens at the UMN VDL and a phylogenetic tree was constructed using NextStrain comparing those with 365 North America GenBank publicly available PRRSV2 WGS. The subset of samples submitted for WGS was selected amongst all samples available by prioritizing samples with Ct values <25, to assure at least one sequence from each production system (if a production system had more than one available sample, the oldest and newest sample were selected), and to include all breeding herd cases available.

In order to investigate if the frequency of risk factors for PRRS occurrence is different in cases (i.e., PRRSV outbreaks of the emerging variant) compared to controls (i.e., PRRSV outbreaks due to other PRRSV variants), a non-matched case-control study was designed based on the MSHMP PRRSV orf5 sequence database. This investigation took place during the first peak in this variant's transmission; thus, a site was eligible to participate in the case-control if it belonged to an MSHMP participant system (due to data accessibility) and had any PRRS sequence generated between October and December 2020. Cases were defined as sites in which the new variant had been detected throughout this period. Controls were defined as any MSHMP participant site that have identified a PRRSV sequence that did not match our case criteria within the same time and space (i.e., Minnesota and Iowa) where cases occurred but also from systems in which this newly emerging variant had been identified. Exclusion criteria comprised sequences with <600 bp and incorrect initial and/or stop codons, since this would influence their probability of being classified as a case. Sites in which more than one sequence was retrieved during the mentioned timeframe that would fit both case and control criteria were also excluded. Because the occurrence of this new variant was heavily clustered in time and space, all eligible cases and controls were included in the study to ensure at least a 1:1 case-control ratio.

A standardized questionnaire regarding known risk factors for PRRSV transmission was applied to each of the involved production systems' leading veterinarians through video calls. Geographic coordinates for each farm, as well as short-term production losses after the PRRS sequence was detected were also requested. A mixed effect logistic regression model was constructed to investigate the odds of being a case given each potential explanatory variable using production system as a random effect. Odds ratio (OR) and 95% confidence interval for each of the variables investigated were estimated based on the number cases and controls exposed. Statistical analyses were conducted using STATA v15.1 (13). Lastly, a purely spatial Bernoulli model for the spatial scan statistics test (14) was performed using circular window with a maximum of 50% of the population at risk with 9999 Monte Carlo simulations using cases and controls to investigate local clustering for cases.

## Results

### Temporal and Spatial Distribution of the Newly Emerging Variant

**Figure 1:**
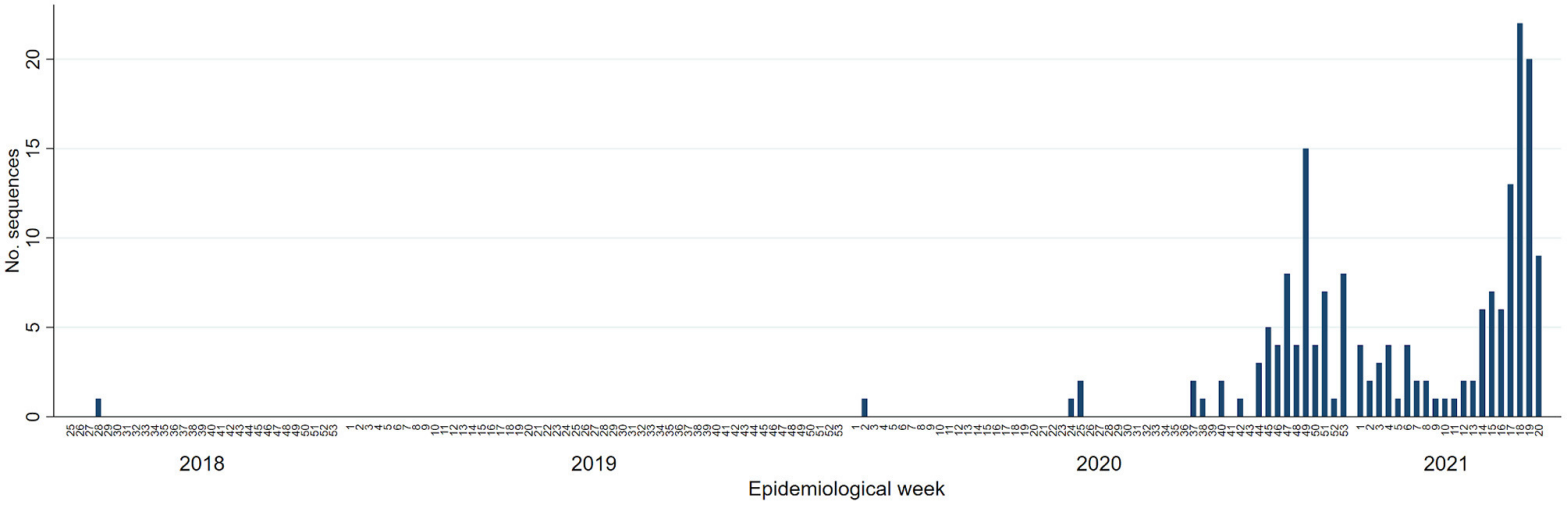
Epidemic curve of the Porcine Reproductive and Respiratory Syndrome Virus Lineage 1C variant associated with this outbreak by epidemiological week amongst participants of the Morrison Swine Health Monitoring Project in the United States, January 2018 to May 2021.

A total of 190 sequences identified between July 2018 and May 2021 were classified as cases of this newly emerging variant. These originated from 154 pig farms (34 breeding, 118 growing pig farms, 11 recoded as other types, and 2 not disclosed) belonging to 14 pig production systems. One sequence was from 2018, 69 were from 2020, 112 were from 2021, and eight had no collection date available. Cases occurred in two waves—the first peak in transmission occurring between October and December 2020 accounted for 61 out of the 190 (32.11%) sequences identified (Figure 1), followed by a decrease between January and March 2021 (*n* = 27, 14.21%) and a second on-going peak was observed in April–May 2021 with 85 (44.74%) sequences identified. The remaining 17 sequences (8.95%) occurred prior to October 2020.

State of origin information was available for 187 of the 190 cases (98.42%), all of which were from the Midwest (145 cases from Minnesota, 40 from Iowa, 1 from Illinois, and 1 from Wisconsin). Coordinates were available for 131 (68.95%) of the cases, and 84.73% (111/131) of those were within a 120 km radius. The average angle of spread was 153.19 degrees (*p* = 0.005), considering the zero on East, and 90 on North.

### Genetic Characteristics of the Newly Emerging Variant

The orf5 sequences of all cases were classified as lineage 1 sub-lineage C (“Lineage 1C”). RFLP type was assigned to 175/190 (91.15%) of the case sequences. Most (*n* = 172; 98.29%) were RFLP 1-4-4 cut pattern, while two were 1-4-3 (one from 2018 and one from 2021) and one was a 1-7-4 (from 2021) RFLP cut pattern. Co-circulation of unrelated PRRSV lineage 1C RFLP pattern 1-4-4 sequences within the same region and time period was observed when contrasting the MSHMP database with case sequences. Those unrelated sequences had a percent nucleotide identity ranging from 91 to 93% to sequences from the cases' genetic cluster.

**Figure 2:**
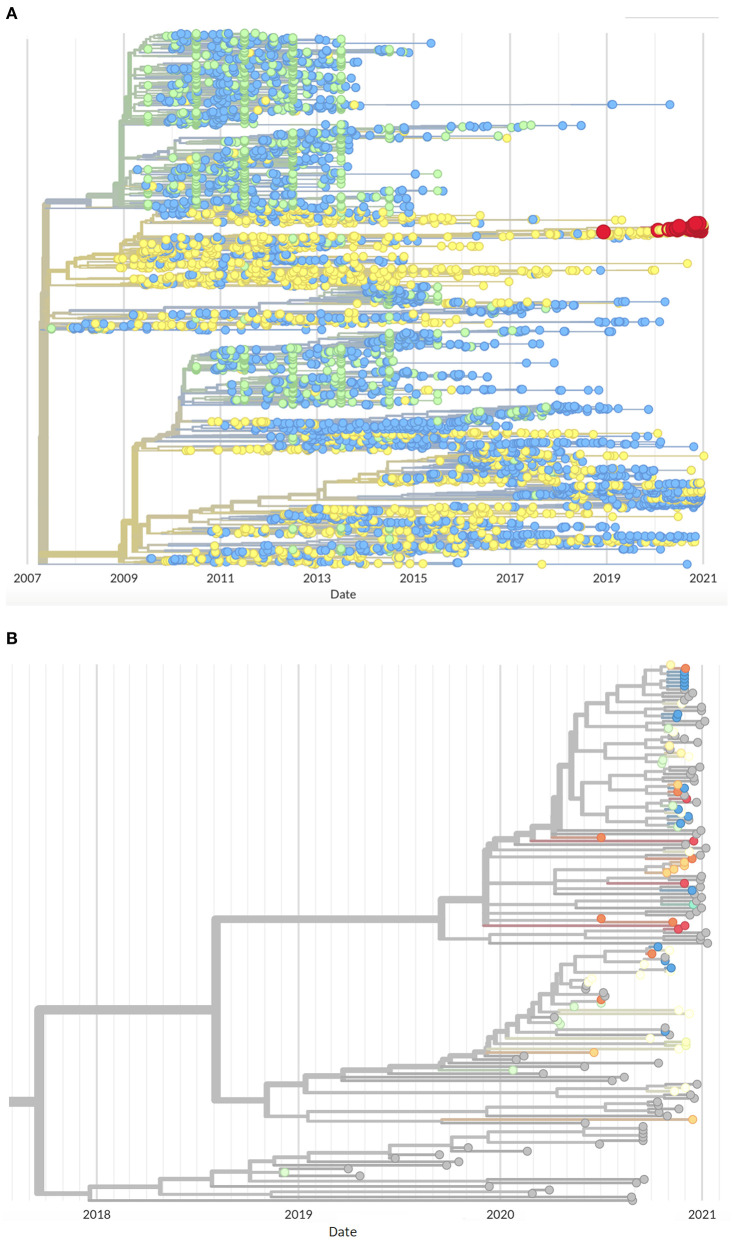
Phylogenetic tree of Lineage 1C orf5 sequences **(A)** Originating from MSHMP (blue), GenBank (green) and the University of Minnesota Veterinary Diagnostic Lab (yellow) PRRS databases showing the outbreak cases highlighted in red, and **(B)** Close up of the same tree showing sequences belonging to this outbreak colored by production systems (gray indicates sequences from the University of Minnesota Veterinary Diagnostic Lab PRRS database).

The orf5 lineage 1C phylogenetic tree constructed (Figure 2A) found an additional 83 sequences (90% from 2020 to January 2021) belonging to the cases' clade with the addition of the UMN VDL database, from which production system information was not disclosed, further emphasizing the emergent nature of this variant. The cases genetic cluster analyzed here does not share a recent ancestor with sequences in any of the evaluated databases since approximately 2018 (Figure 2B), suggesting that this event represents the emergence of a new variant within Lineage 1C distinct from previously circulating L1C sequences.

Accuracy of the different PRRSV2 classifications is described in Table 1. While all three classifications assessed had sensitivities, specificities, and negative predictive values of ≥90%, a huge difference was observed in their positive predictive values. The classification with the lowest positive predictive value was RFLP pattern 1-4-4, with only 34%, followed by Lineage 1C classification with 43%. The combination of both classification methods (Lineage 1C RFLP pattern 1-4-4) provided a large increase in the positive predictive value to 81%.

**Table 1:** Accuracy of RFLP pattern (1-4-4), lineage classification (Lineage 1C), and the combination of both in identifying sequences from the newly emerging variant amongst PRRSV orf5 sequences detected between January 2020 and May 2021.

**Classification**	**TP**	**FP**	**FN**	**TN**	**% Sensitivity (95%CI)**	**% Specificity (95% CI)**	**% Positive predictive value (95% CI)**	**% Negative predictive value (95% VI)**
RFLP pattern 1-4-4	171	18	339	3149	90.48 (85.37–94.26)	90.28 (89.25–91.24)	33.53 (29.44–37.81)	99.43 (99.10–99.66)
Lineage 1C	189	0	257	3231	100 (98.07–100)	92.63 (91.71–93.48)	42.38 (37.74–47.11)	100 (99.89–100)
Lineage 1C RFLP pattern 1-4-4	171	18	41	3447	90.48 (85.37–94.26)	98.82 (98.41–99.16)	80.66 (74.69–85.75)	99.48 (99.18–99.69)

**Figure 3:**
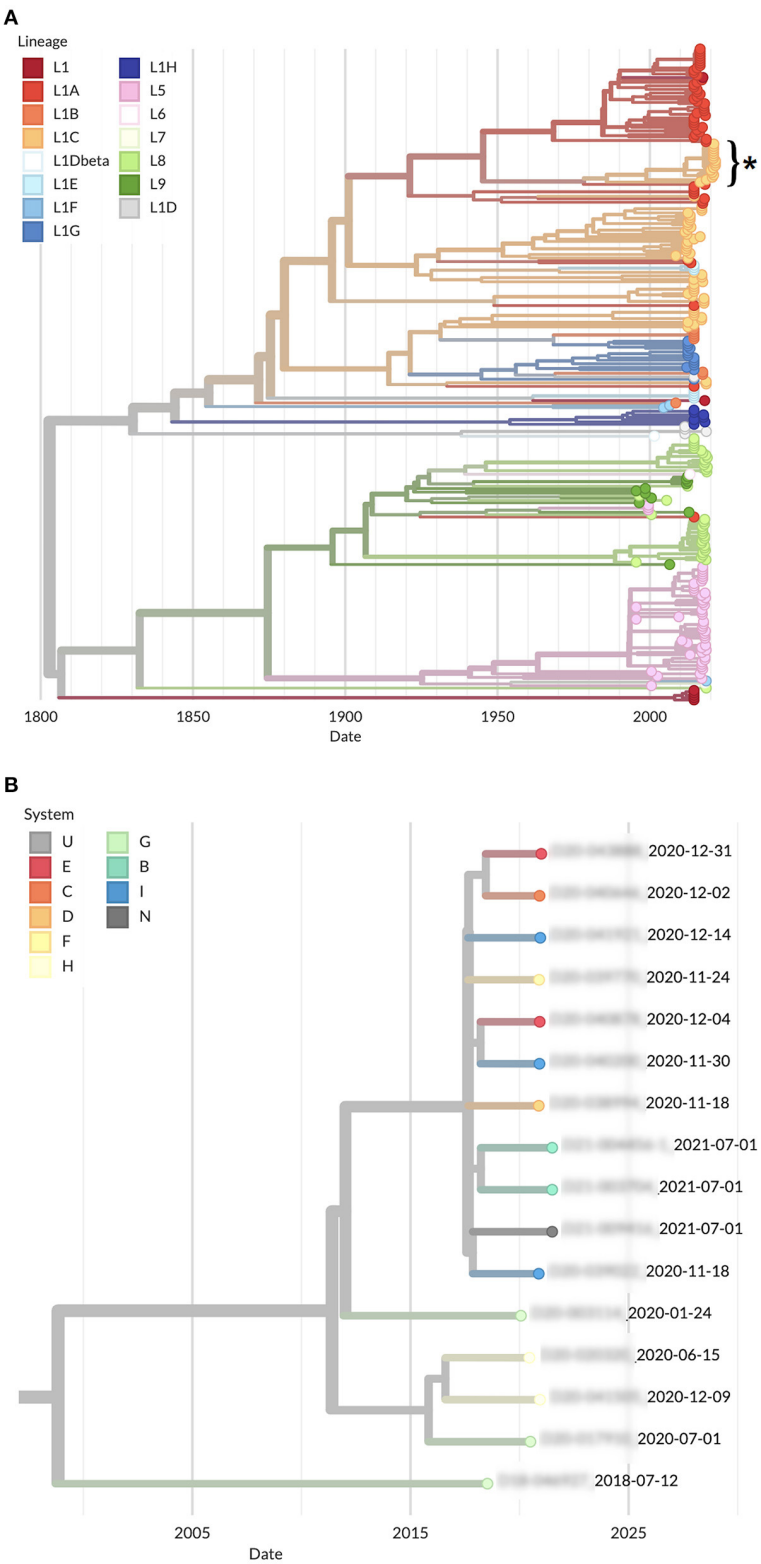
Phylogenetic tree of PRRSV whole genome sequences **(A)** Publicly available at GenBank color coded by lineage (asterisk represents outbreak cases), and **(B)** Close up of the clade containing all sequences belonging to this outbreak color coded by production system.

When comparing the subset of 16 WGS to sequences available in GenBank, all cases remained clustered within one single separate clade (Figure 3). The highest similarity between the consensus WGS from all cases and all GenBank PRRSV WGS was 99.89% to MW887655.1, a sequence generated after these cases began occurring in December 2020. All other WGS publicly available had ≤ 95% identity to the WGS consensus sequence from cases. Amongst the 16 whole genome sequences from this newly emerging variant obtained, their nucleotide identity in orf1 ranged from 96.33% to 99.85%, from 97.93% to 100% in orf2a, 96.85% to 100% in orf2b, 97.12% to 100% in orf3, 94.97% to 100% in orf4, 97.68% to 100% in orf5, 98.86% to 100% in orf6, and 98.66% to 100% in orf7. Overall, percent nucleotide identity between all 16 whole genomes ranged from 96.28% to 99.82%.

### Case-Control Investigation

A total of 50 cases and 58 controls farm-level PRRS outbreaks that occurred between October and December 2020 from nine production systems were included in this study. Among cases, nine (18.00%) were breeding herds and 41 (82.00%) were grow-finishing herds, while nine (15.52%) controls were breeding herds and 49 (84.48%) were grow-finishing herds (chi-square *p* = 0.73). Consistent with the overall description of sites affected with this variant, most cases (45/50; 90.00%) occurred in Minnesota, while five (10.00%) occurred in Iowa. However, 60.34% (35/58) of the selected controls were from Iowa, the remaining being from in Minnesota (chi-square *p* < 0.001).

**Figure 4:**
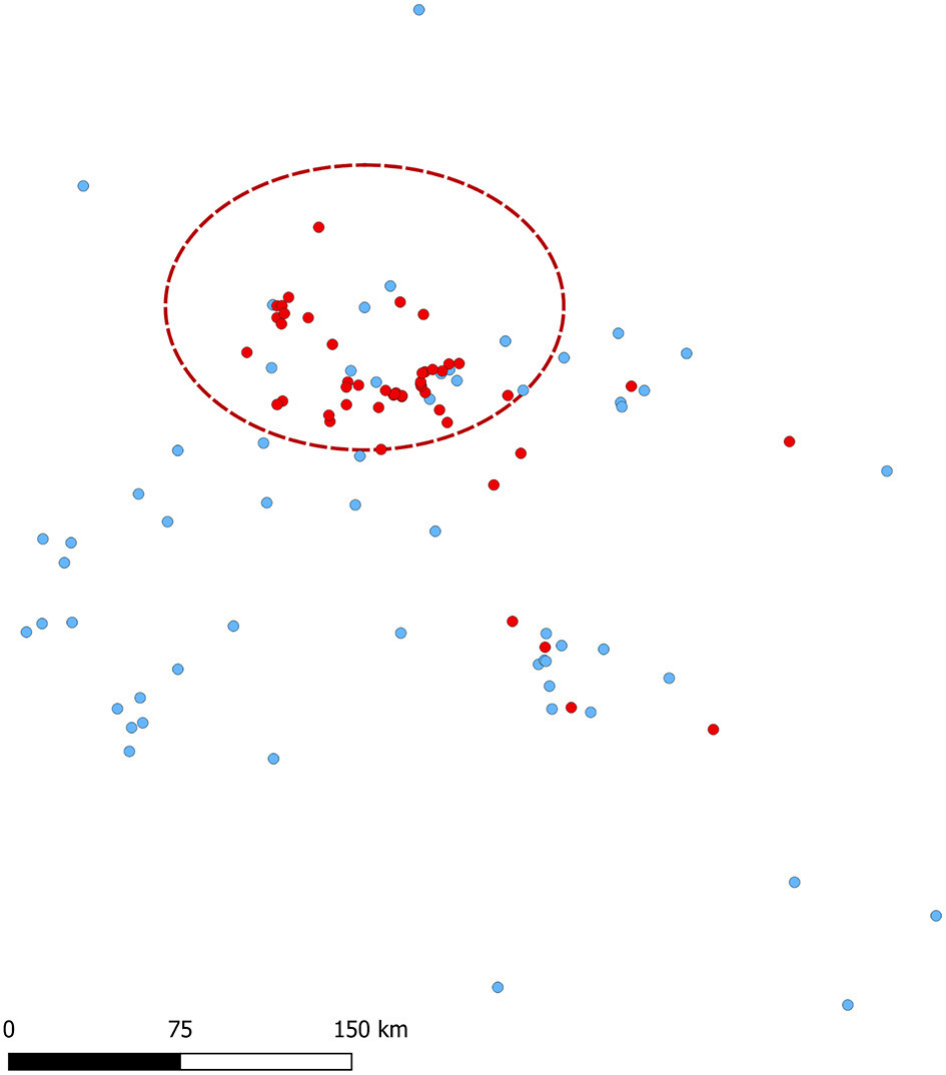
Cases (red) and controls (blue) spatial distribution and spatial clustering of cases (red dashed ellipse).

Sites that had a market vehicle that was not exclusive to the system had 0.04 times the odds of being a case than a control (Table 2). Among sites that use rendering as mortality management, information on rendering company was available for 84.62% (55/65), most of which used the same company *X* (45/55; 81.82%). The proportion of sites that used company *X* for rendering did not differ between cases (18/24; 75.00%) and controls (27/31; 87.10%; chi-square *p* = 0.32). Among the 18 breeding sites in the study (nine cases and nine controls), gilt acclimation was done with live virus inoculation (LVI) in three sites (two cases and one control), commercial vaccines were used in seven (three cases and four controls), both LVI and commercial vaccines were used in one control site, and no gilt acclimation was done in six sites (four cases and two controls). Feed source information was available for 48 controls and 44 cases, with sites sourcing from 26 distinct locations. The odds of sourcing from each specific feed mill location were not statistically different among cases than among controls. Additionally, the use of feed mitigants was not statistically different between cases and controls (*p* = 0.88; Table 2). Of the 50 vaccinated herds, 20 were cases and 30 were controls. The production systems used four different vaccines: Ingelvac PRRS MLV (Boehringer Ingelheim Animal Health, Duluth, Georgia, USA), PrimePac PRRS (Merck Animal Health, Kenilworth, New Jersey, USA), Prevacent PRRS (Greenfield, Indiana, USA), or Fostera PRRS (Zoetis, Parsipanny, New Jersey, USA). The 20 vaccinated cases comprised two breeding sites (one using Ingelvac MLV and one using PrimePac for gilt acclimation) and 18 growing pig sites (nine using Prevacent, six using Ingelvac MLV, two using PrimePac, and one using Fostera). The 30 vaccinated controls comprised five breeding sites (four using Ingelvac MLV and one using Prevacent for gilt acclimation) and 25 growing pig sites (11 using Fostera, seven using Ingelvac MLV, and seven using Prevacent). No statistical difference in vaccination status or vaccine used was found between cases and controls. Noteworthy, the frequency of sites with air filtration did not differ between cases and controls (*p* = 0.25, Table 2). A geographical cluster with 81.42 km radius was identified in which the number of cases observed was 1.68 times higher than expected compared to controls (*p* = 0.0001; Figure 4).

**Table 2:** Factors associated with PRRSV transmission between within herd outbreaks caused by the newly emerging Lineage 1C variant (cases) and other PRRSV variants (controls).

	**Case**		**Control**		**OR (95%CI)**	***p*** **-value**
	***n***	**%**	***n***	**%**		
**Feed vehicle**						
Exclusive to the system	7	17.07	8	15.09	1	–
Not exclusive	34	82.93	45	84.91	0.57 (0.04–8.68)	0.69
**Use of feed mitigant**						
Yes	8	19.51	7	12.5	0.87 (0.16–4.87)	0.88
No	33	80.49	49	85.5	1	–
**Dead animals management**						
Compost on-site	21	43.75	15	27.27	1	–
Incinerated on-site	1	2.08	1	1.82	–	–
Rendering	26	54.17	39	70.91	0.49 (0.13–1.82)	0.29
**Manure storage**						
Deep pit	42	85.71	50	86.21	1	–
Lagoon	7	14.29	7	12.07	1.15 (0.24–5.50)	0.86
Deep pit and lagoon	0	0	1	1.72	–	–
**Air filtration**						
Not filtered	47	94	54	93.1	1	
Filtered	3	6	4	6.9	0.30 (0.04–2.38)	0.25
**Site had maintenance in the month prior to sequence detection**						
No	19	86.36	32	94.14	1	–
Yes	3	13.64	2	5.88	2.84 (0.12–66.67)	0.52
**Site has tree windbreaks**						
No	26	65	35	76.09	1	–
Yes	14	35	11	23.91	1.70 (0.40–7.26)	0.48
**Animals drinking water source**						
Rural water	2	4.08	3	5.45	1	–
Well	47	95.92	52	94.55	0.18 (0.02–1.83)	0.15
**Is water treated?**						
No	42	97.67	53	92.98	1	–
Yes	1	2.33	4	7.02	0.32 (0.02–6.41)	0.46
**Was the herd vaccinated**						
No	30	60	28	48.28	1	–
Yes	20	40	30	51.72	0.51 (0.13–1.99)	0.33
**Site has a disinfection and drying room**						
No	36	87.8	48	88.89	1	–
Yes	5	12.2	6	11.11	0.23 (0.02–2.49)	0.23
**Is personnel shared with other sites?**						
No	19	44.19	15	30	1	–
Yes	24	55.81	35	70	1.07 (0.34–3.32)	0.91
**Age of animals when sequence was detected**						
<10 weeks	19	63.33	27	71.05	1	–
≥10 weeks	11	36.67	11	28.95	1.63 (0.43–6.14)	0.47
**Was the site double stocked?**						
No	31	83.78	33	67.55	1	–
Yes	6	16.22	16	32.65	0.57 (0.15–2.20)	0.42
**Was it an all-in-all-out site?**						
No	10	31.25	6	13.33	1	–
Yes	22	68.75	39	86.67	0.30 (0.04–2.41)	0.26
**Market vehicle exclusivity to the system**						
Exclusive	13	44.83	1	3.03	1	–
Not exclusive	16	55.17	32	96.97	0.04 (0.00–0.43)	0.01
**Did the site loaded or unloaded animals in the two weeks prior to detecting the sequence?**						
No	22	70.97	22	57.89	1	–
Yes	9	29.03	16	42.11	0.41 (0.11–1.50)	0.18
**Did the site receive animals form a positive sow farm?**						
No	27	77.14	32	82.05	1	–
Yes	8	22.86	7	17.95	1.08 (0.20–5.80)	0.93

Information on the average nursery or finishing mortality rates within the first 4 weeks after detection of the farm-level PRRS outbreak was obtained for 11 cases and 27 controls from three production systems. Cases had a median nursery mortality of 0.11% (IQR: 0.07–0.21%, range: 0.05–7.25%) while controls had median mortality of 0.02% (IQR: 0.01–0.81%, range: 0.01–5.23%; Wilcoxon rank-sum *p* = 0.08). Similarly, the cases' median finishing mortality was 4.50% (IQR: 0.04–8.80%, range: 0.01–11.25%) while controls' was 0.01% (IQR: 0.01–0.02%, range: 0.003–1.08%; Wilcoxon rank-sum *p* = 0.01).

## Discussion

The magnitude of this emerging outbreak is likely underestimated despite our efforts to collect sequences from varied sources. This was evidenced by the additional cases detected with the addition of the UMN VDL sequence dataset. However, because MSHMP monitors ~50% of the U.S. breeding herd and because of access to all participant's breeding, gilt developing units, growing, and finishing herds sequencing information, we believe representativeness is good for the description of sites affected. PRRS occurrence in breeding herds has been demonstrated to have a seasonal pattern, in which the majority of farm-level outbreaks occurs during the fall and winter, with few cases during spring and summer (15). Still, while the Midwest U.S. seem to have been the only heavily affected region by this PRRSV variant at the time of writing, the fact that transmission carried on throughout the spring, with the number of cases surpassing the observed during winter, rises concerns for the next high transmission season of PRRS.

The high similarity between the viruses, as well as their clustered occurrence in both time and space affecting several production systems characterizes this as a region-wide outbreak. The occurrence of farm-level PRRS outbreaks in several sites associated with such a highly similar virus at the orf5 region is unprecedented. PRRSV2 Lineage 1C was found to be prevalent in one region of the U.S. *circa* 2009–2014 (5). Yet, sequences associated with this region-wide outbreak correspond to a unique clade within Lineage 1C. The orf5 gene has been used as an industry standard for PRRSV2 identification since it encodes for the major envelope glycoprotein (GP5), which plays a role in inducing virus neutralizing antibodies and cross-protection among PRRSV variants (16, 17). It also represents one of the most genetically variable regions in the PRRSV genome (18). The PRRSV whole genome sequence database is still scarce and its representativeness might pose limitation when contextualizing cases associated with this outbreak. While by mid-February 2021 ~1,200 PRRS whole genome sequences were available in GenBank, this figure raised to over 26,500 considering PRRSV orf5 sequences. Additionally, whole genome sequencing is currently not performed as a surveillance tool in the swine industry. While PRRSV orf5 sequencing may also be biased toward a combination of low cycle threshold (ct) samples, virulent strains or outbreak investigations, PRRSV whole genome sequencing might be biased toward an even more restricted criteria such as when orf5 results are conflicting or unexpected. Results found in both our orf5 and WGS analyzes further corroborate that this is likely the emergence of a new PRRSV variant, a sub-clade within Lineage 1C.

The emergence of this new clade within Lineage 1C, paired with the important clinical manifestations reported from industry stakeholders, has significance to the swine industry. No particular known risk factors for PRRS other than the market vehicle being exclusive to the production system were found to be associated with the transmission for this variant compared to other PRRSV variants. This suggests that this variant might be more easily spread within production systems as a consequence of potentially improved viral properties but that the overall risk factors for PRRSV transmission remain the same. Most sites included both as cases and controls were growing pig sites, in which biosecurity practices are not as strict as in breeding herd sites. Reports from the field suggest that Ct values of positive samples to this variant tend to be lower than for other PRRSV variants (19). This was not assessed in this study and remains to be confirmed under experimental and more controlled conditions. However, if true, a higher environmental contamination due to higher viremia and viral shedding could explain the lack of association of a particular risk factor to the transmission of this variant. Experimental studies are warranted to investigate this hypothesis. Additionally, even though all cases and all controls that fit our inclusion and exclusion criteria were used in this study, we might still lack power to detect smaller differences in frequency of exposures. Additional field and experimental investigations are still needed to fully understand how this virus is spreading.

Our study indicates that the average finishing mortality in the first 4 weeks is higher in herds affected with this variant. An important limitation here is that we were not able to obtain mortality information for all cases and controls. Personal communications from field veterinarians managing affected sites described weekly pre-weaning mortality of up to 100% in breeding herds, and wean-to-market mortality up to 40% in growing pigs. However, some experienced much lower production impact. The mortality data captured in our study has a smaller range, but still showed differences between sites affected by this variant compared to others. Many factors may influence mortality rates in the grow-finishing phase and were not assessed in the present study, such as co-infections, management, and weaning age (20). Thus, a more comprehensive understanding of this variant's virulence is still warranted.

Lastly, our findings suggest that the case definition for this outbreak should not rely solely on RFLP types or lineage and instead contextualize sequences through a phylogenetic approach. Viral characterization and a case definition beyond RFLP patterns and lineage classification is warranted as similar co-circulating viruses can mislead data interpretation at the field level. An assessment of the most commonly used PRRSV2 classifications to describe this newly emerging variant was made in this study. Our results show low positive predictive values for either RFLP pattern 1-4-4 or Lineage 1C, suggesting there is a low probability of a sequence classified as any of these definitions to actually comprise the newly emerging variant described here. Although a combination of both classification methods highly increases the positive predictive value, noteworthy, it does not have a perfect predictive value. This is relevant particularly to field veterinarians and producers who might receive this information from the VDL upon submitting a sample for PRRSV orf5 sequencing. Additionally, it is also relevant for communications regarding this particular variant to assure a correct case definition. Currently, there is no standardized classification criteria for variant calling at this level of information regarding PRRSV orf5 sequences. Similarly, there is no standardized criteria for variant calling for PRRSV whole genome sequences. Here, we used lineage classification to color whole genome phylogeny tree nodes. However, lineage classification is orf5 based and thus the genetic relationships used for its definition might not hold true when assessing the remaining parts of the genome. There is clearly a need for revisions and standardization of variant calling for PRRSV2 in a level of detail that would benefit epidemiological investigations. For the purposes of identifying new cases potentially associated with this newly emerging variant, a subset of orf5 sequences representative of 83% of cases from this outbreak (68% of the genetic diversity) up to December 2020 are deposited at GenBank (accession numbers MW525326-MW525341, MW525343). Although this first epidemiological description serves as an industry alert of an ongoing regional outbreak with a newly emerged variant, additional virological and epidemiological studies are warranted to fully understand this variant's transmissibility, *ex-vivo* survivability, pathogenicity and immunogenicity.

## Data Availability Statement

The data that support the findings of this study are available upon request to the corresponding author. The data are not publicly available due to privacy restrictions with the exception of selected orf5 sequences made publicly available at GenBank (https://www.ncbi.nlm.nih.gov/genbank/), accession numbers MW525326-MW525341, MW525343.

## Author Contributions

MK and CC contributed to conception and design of the study. MK, IP, NP, and CP-R performed data analysis. MK wrote the first draft of the manuscript. CC, KV, and AR supervised the findings of this work. MS, PY, BL, LB, DM, BR, PT, LF, MA, MH, TB, and BS brought this issue to attention and provided all data for the analysis. All authors contributed to manuscript revision, read, and approved the submitted version.

## Funding

This project was funded by the Swine Health Information Center (SHIC, www.swinehealth.org), Project # 19-235 SHIC (CC, MK, and CP-R), University of Minnesota Swine Disease Eradication Center - PRRS 144 Whole Genome Sequence Study (CC, MK, and NP) and the joint NIFA-NSF-NIH Ecology and Evolution of Infectious Disease (www.nsf.gov), award 2019-67015-29918 (KV, IP, and NP). The funders had no role in study design, data collection and analysis, decision to publish, or preparation of the manuscript.

## Conflict of Interest

MS is employed by Schwartz Farms Inc. PY, BL, and LB are employed by Swine Vet Center. DM is employed by New Fashion Pork. BR is employed by Fairmont Veterinary Clinic. PT is employed by Iowa Select Farms. LF is employed by Protein Sources Management. MA is employed by Holden Farms Inc. MH, TB, and BS are employed by The Maschhoffs LLC. The remaining authors declare that the research was conducted in the absence of any commercial or financial relationships that could be construed as a potential conflict of interest.

## Publisher's Note

All claims expressed in this article are solely those of the authors and do not necessarily represent those of their affiliated organizations, or those of the publisher, the editors and the reviewers. Any product that may be evaluated in this article, or claim that may be made by its manufacturer, is not guaranteed or endorsed by the publisher.
